# Meta-analysis of cell- specific transcriptomic data using fuzzy c-means clustering discovers versatile viral responsive genes

**DOI:** 10.1186/s12859-017-1669-x

**Published:** 2017-06-06

**Authors:** Atif Khan, Dejan Katanic, Juilee Thakar

**Affiliations:** 1 Department of Microbiology and Immunology, University of Rochester, Rochester, NY 14642 USA; 2 Department of Biostatistics and Computational Biology, University of Rochester, Rochester, NY 14642 USA; 3 601 Elmwood Avenue, Rochester, NY 14618 USA

**Keywords:** Epithelial cells, Dendritic cells, Gene-sets, Influenza infections, Gene-gene mutual information, Overlapping gene-sets

## Abstract

**Background:**

Despite advances in the gene-set enrichment analysis methods; inadequate definitions of gene-sets cause a major limitation in the discovery of novel biological processes from the transcriptomic datasets. Typically, gene-sets are obtained from publicly available pathway databases, which contain generalized definitions frequently derived by manual curation. Recently unsupervised clustering algorithms have been proposed to identify gene-sets from transcriptomics datasets deposited in public domain. These data-driven definitions of the gene-sets can be context-specific revealing novel biological mechanisms. However, the previously proposed algorithms for identification of data-driven gene-sets are based on hard clustering which do not allow overlap across clusters, a characteristic that is predominantly observed across biological pathways.

**Results:**

We developed a pipeline using fuzzy-C-means (FCM) soft clustering approach to identify gene-sets which recapitulates topological characteristics of biological pathways. Specifically, we apply our pipeline to derive gene-sets from transcriptomic data measuring response of monocyte derived dendritic cells and A549 epithelial cells to influenza infections. Our approach apply Ward’s method for the selection of initial conditions, optimize parameters of FCM algorithm for human cell-specific transcriptomic data and identify robust gene-sets along with versatile viral responsive genes.

**Conclusion:**

We validate our gene-sets and demonstrate that by identifying genes associated with multiple gene-sets, FCM clustering algorithm significantly improves interpretation of transcriptomic data facilitating investigation of novel biological processes by leveraging on transcriptomic data available in the public domain. We develop an interactive ‘Fuzzy Inference of Gene-sets (FIGS)’ package (GitHub: https://github.com/Thakar-Lab/FIGS) to facilitate use of of pipeline. Future extension of FIGS across different immune cell-types will improve mechanistic investigation followed by high-throughput omics studies.

**Electronic supplementary material:**

The online version of this article (doi:10.1186/s12859-017-1669-x) contains supplementary material, which is available to authorized users.

## Background

Microarrays and RNA-seq have made simultaneous expression profiling of many thousands of genes across several experimental/clinical conditions widely accessible. However, interpreting the profiles from such large numbers of genes remains a key challenge. An important conceptual advance in this area was a shift from a focus on differential expression of single genes to testing sets of biologically related genes [1–5]. Gene-sets are defined a priori as sharing some biologically relevant properties (e.g. members of the same pathway, having a common biological function, presence of a binding motif, etc.). In addition to the obvious advantage in interpretability, a key benefit of analyzing gene-sets compared with individual genes is that small changes in gene expression are unlikely to be captured by conventional single-gene approaches, especially after correction for multiple testing [1].

Despite advances in the methods for gene-set enrichment analysis [2, 6–8]; inadequate definitions of gene-sets cause a major limitation in the discovery of novel biological processes. Typically, gene-sets are obtained from pathway databases available in the public domain such as Kyoto Encyclopedia of Genes and Genomes (KEGG). However, recent advances have led to development of data-driven approaches to identify gene-sets [9–13]. These are powerful approaches that expand search for biological mechanisms based on datasets in public domain leading path towards discovery.

**Fig. 1:**
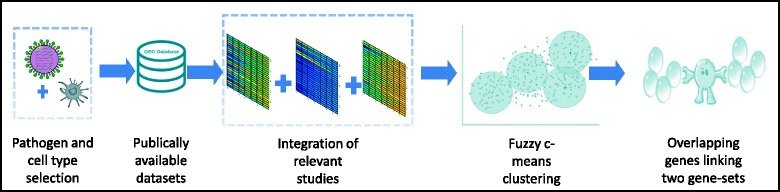
Schematic representation of FIGS pipeline. The context-specific datasets obtained from public repositories were integrated as described in [20]. FCM is performed on gene-gene mutual information matrix. Gene-sets obtained from optimized FCM clustering were compared with KEGG pathways for validation and multi-functional genes connecting different gene-sets were identified

The data-driven identification of gene-sets is performed by measuring pair-wise co-expressions or association between genes which is followed by different, typically unsupervised hard (such as K-means and hierarchical) clustering approaches [14–17]. However, there are two limitations- first, biological pathways show a large overlap pertaining to the modular structure of signal transduction processes which is not reproduced by hard clustering algorithms and second, functional interpretation of novel gene-sets is difficult if they are not enriched in any known pathways. Here we propose a computational pipeline (Fig. 1) based on Fuzzy C-Means (FCM) clustering method [18, 19] which allows overlap across gene-sets, thus reproducing the observed topology of biological pathways, and associate novel gene-sets to other gene-sets with enrichment of known pathways. Particularly, we apply the FCM pipeline to our previously curated context-specific data [20]. To facilitate use of our pipeline we developed a downloadable ‘Fuzzy Inference of Gene-sets (FIGS)’ package available at GitHub (https://github.com/Thakar-Lab/FIGS). Here, we demonstrate its application using transcriptomic data obtained from Gene Expression Omnibus (GEO) measuring response to infections of monocyte derived dendritic cells (DC) and A549 epithelial cells (EC) with influenza virus [20]. The gene-sets and overlapping genes identified in this study are validated by assessing enrichments of known pathway genes. Thus, robust data-driven gene-sets identified by FIGS retain the characteristics of known pathways and expand the search of new mechanisms.

## Methods

### Datasets

Transcriptomic data was obtained from GEO and was integrated in cell-specific manner. Integration procedure and calculations of associations between genes has been described in detail previously [20]. Briefly, transcriptomic data measuring changes in gene-expression in monocyte derived dendritic cells (DC) and A549 epithelial cells (EC) upon influenza infections were used. There were two datasets for DCs (GSE41067 and GSE55278) and 9 datasets for ECs (GSE19580, GSE31469, GSE31470, GSE31471, GSE31472, GSE31473, GSE31474, GSE31518 and GSE47937). All the datasets were log2 transformed and quantile normalized individually in a platform specific manner as described previously [20]. To facilitate comparison across independent datasets, 14,894 genes commonly present across all the studies were used in this analysis. Fold changes in influenza infected samples were calculated relative to the non-infected samples and genes with absolute fold change > 1 in atleast one sample were kept. After this filtration, 3846 and 5789 genes were present in EC and DC dataset respectively. Mutual information (MI) was calculated to describe the associations between 3846 and 5789 genes within EC and DC respectively [20–22]. The computational pipeline proposed below was developed on DC data and was applied to EC data. Moreover, for comparison and validation of our method we used filtered set of immunologically relevant pathways from Kyoto Encyclopedia of Genes and Genomes (KEGG) [8, 23].

### Soft and hard unsupervised cluster analysis

To assess the usability of the FCM clustering to identify gene-sets, it was compared with previously used hard clustering approaches [20]. Particularly, k-means clustering [24, 25] was performed with the following objective function:

1 $$ J={\displaystyle \sum_{k=1}^K}\ {\displaystyle \sum_{i\varepsilon Ck}}{\left\Vert {\mathrm{x}}_{\mathrm{i}}-{\mu}_k\right\Vert}^2 $$

Where, µ_*k*_ is the centroid of the *k*_*th*_ cluster and *x*_*i*_ is the *i*_*th*_ observation.

Unlike hard clustering techniques, FCM method [18, 19] allows a data point to belong to multiple clusters. FCM is a soft version of k-means, where each data point has a fuzzy degree of belonging to each cluster. The fuzzy degree of belongingness ranges from 0 to 1 where 0 shows no association and 1 shows complete association of a data point to the corresponding cluster. The FCM was performed with the following objective function:

2 $$ J={\displaystyle \sum_{i=1}^{n\kern0.5em }}\ {\displaystyle \sum_{j=1}^{\kern0.5em  c}}{w}_{i j}^m{\left\Vert {x}_i-{c}_j\ \right\Vert}^2 $$

Where,

3 $$ {w}_{i j}^m=\frac{1}{{\displaystyle {\sum}_c^{k=1}}{\left(\frac{\left\Vert {x}_i-{c}_j\right\Vert }{\left\Vert {x}_i-{c}_k\right\Vert}\right)}^{\frac{2}{m-1}}} $$

Thus, FCM algorithm assigned genes to one or more clusters with different degrees of memberships.

### Optimization of fuzzy c-means clustering parameters

Determination of the initial number of clusters is a question of ongoing debate, especially when overlap (fuzzy) across clusters is expected [26]. We determine the initial number of clusters (﻿*K﻿*) by taking into account average number of genes per cluster based on known pathways and the underlying structure of data from principal component analysis (PCA) [27]. Specifically, for DC and EC first 50 principal components explained >90% of the total variance. Hence, we used equivalent (50) number of clusters for the following analysis. Note that, the algorithm could converge to a different number of clusters, than what had been defined initially. These clusters are referred to as gene-sets in the results section due to their usability in gene-set enrichment analysis.

**Fig. 2:**
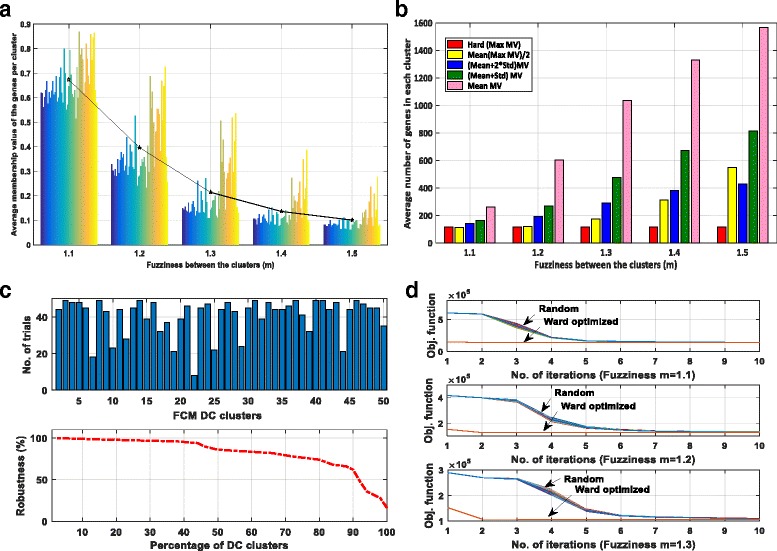
Optimization of FCM parameters. **a** Average membership value (*y-axis*) per cluster with increasing fuzziness (*x-axis*), **b** Average number of genes per cluster (*y-axis*) for increasing fuzziness (*x-axis*) and four cluster association criteria, **c** 50 trials conducted with random initial assignment of the centroids found only 16% reproducible clusters, **d** Objective function values for FCM clustering with initial centroid assignment performed randomly  and by Ward’s method (*red line*) under fuzziness 1.1, 1.2 and 1.3 respectively. Ward based initialization converged more rapidly and produced stable and robust clustering solution

FCM requires three additional pre-defined parameters: fuzziness (the amount of overlap between the clusters), initial cluster centroids and cluster association criteria which is specific to the data distribution [28]. The fuzziness and cluster association are inversely related since fuzziness defines the belongingness of the genes to specific clusters. Thus, the selection of fuzziness and the clusters’ association determines the size and amount of overlap between the clusters. Here, the objective was to identify the functionally related genes which typically range from 100 to 500 depending on the biological process [29]. The length of 45 selected immunologically relevant KEGG pathways ranged from 23 to 362 with an average of 100 genes. Fuzziness (m) ranging from 1.1 to 1.5 was evaluated. Fuzziness *m* = 1.1 preserved strong primary association of a gene to one cluster and intermediate association to another (Fig. 2a). With *m* > 1.1, the average membership value per cluster decreased thus increasing the uncertainty in gene-sets (Fig. 2a). Also, the size of the clusters increased with *m* (Fig. 2b), making functional interpretations difficult. Thus, in the following analysis fuzziness *(m)* was set to 1.1.

The threshold for associating genes to the clusters was determined by evaluating distribution of membership values of genes across 50 clusters. Specifically, the *i*_*th*_ gene *g*_*i*_ belonged to the clusters for which it had membership values greater than (*μ*_*i*_ *+ σ*_*i*_ *)*, where μ_i_ and σ_i_ are mean and standard deviation of membership values of *g*_*i*_ respectively.

### Ward’s minimum variance assigns robust initial cluster centroids

Typically, random initial assignment of the cluster centroids is used in FCM algorithms [28, 30]. However, previous studies and our analysis shows that random initialization leads to inconsistent and unreliable clustering results [31, 32]. In our analysis, only 16% of the clusters were consistent across all 50 iterations of the FCM upon random initialization of the centroids (Fig. 2c). The variation in clustering solutions across 50 iterations showed that FCM is sensitive to initial assignment of the cluster centers and that solution frequently converged at local minima instead of finding the global optimal solution. To overcome this problem, Ward’s minimum variance method was used to estimate the initial centers for FCM which produced stable and consistent clusters [33]. Ward’s method (based on analysis of variance) minimized the total within-cluster variance and maximized between-clusters variance. Cluster membership was evaluated by calculating the total sum of squared deviations from the mean of a cluster. At the initial step, all clusters were singletons (each cluster containing a single gene), which were merged in each next step so that the merging contributed least to the variance criterion. This distance measure called the Ward distance was defined by:

4 $$ {d}_{a b} = \frac{n_a\ .\kern0.5em {n}_b}{n_a + {n}_b}.\ \left\Vert \overline{x_{a\ }} - \overline{x_{b\ }}\right\Vert 2 $$

Where *a* and *b* denote two specific clusters, *n*_*a*_ and *n*_*b*_ denote the number of data points in the two clusters. $\overline{x_a}$ and $\overline{x_b}$ denote the cluster centroids and ‖‖ is the Euclidean norm.

Ward’s method produced hierarchical cluster tree that was cut to produce 50 hard clusters where each gene was fully associated to a unique cluster. The centroids of these 50 clusters were calculated and used for FCM initialization. It was found that the objective function of Ward-optimized FCM solution not only converged faster than that of randomly assigned initial centroids (Fig. 2d) but also provided a stable clustering solution.

### Cluster validation and enrichment with KEGG pathways

The clusters of genes identified by FIGS were tested for their cohesiveness and biological significance. To test the cohesiveness of the clusters a weighted clustering coefficient (CC) was measured. CC provided a measure of the degree of relatedness between the genes in a cluster. The tendency of genes in the cluster to tightly knit groups was estimated by a ratio of mean﻿﻿s of﻿ CCs calculated using only genes in the cluster over all the genes [34, 35]. CC was calculated using functions from gaimc library in MATLAB. The ratios were compared for k-means, Ward’s hierarchical method, and FCM solutions.

We expect that the clusters of genes identified in this study are to be functionally related. In other words, genes belonging to the same pathways were expected to group together. To test this hypothesis, we evaluated whether genes belonging to a same known immunologically relevant pathway cluster together [36]. A set of 44 immunologically relevant pathways obtained from KEGG database along with interferon stimulated genes set (ISGs) defined by Schoggins [37, 38] were compared with the clusters identified by FCM pipeline using hypergeometric test [39, 40].

## Results

### Identification of the gene-sets by FCM

**Fig. 3:**
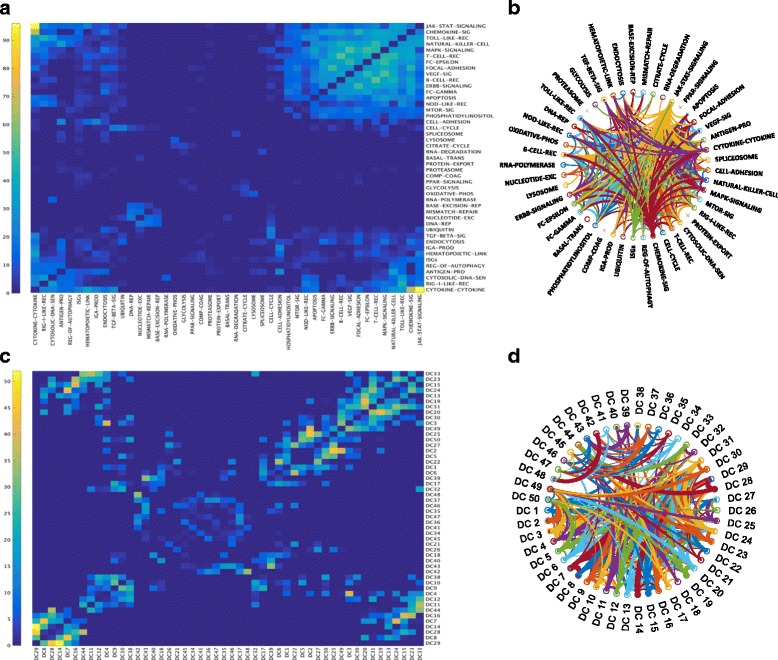
Overlap observed among KEGG pathways and FCM gene-sets. The overlap among KEGG pathways ﻿represented by **a** heat-map **b** circular graph and the overlap among DC FCM gene-sets ﻿represented by **c** heat-map **d **circular graph. The color scale ranging from *blue* to *yellow* in the heat-map (**a**, **c**) and the increasing width of arc (**b**, **d**) correspond low to high number of overlapping genes across pairs of clusters

Signalling pathways from public repositories are generalized static instances of cascades that are frequently derived by curation. Increasing use of high-throughput assays in the biomedical field allows identification of context-specific set of functionally related genes, which can be loosely defined to include genes regulated by a same set of transcription factors or sets of genes involved in same pathways. Recently, use of clustering algorithms has been proposed to identify the “functionally related genes” or gene-modules from publicly available transcriptomics datasets [11, 12, 41]. However, frequently used algorithms such as K-means and hierarchical clustering, for this purpose do not allow overlap between the clusters (referred as gene-sets in rest of the manuscript), although such overlap between biological pathways is inevitable given modular topology of biological response [42]. Specifically, 44 immunologically relevant pathways from KEGG databases suggest a minimum of 0% and maximum of 63% overlap between the two pathways (Fig. 3a). For example, Cytokine-Cytokine receptor interaction and JAK-STAT signaling pathways have 96 genes in common. Interestingly, some genes like AKT1, MAPK1, PIK3CA, and TNF were found involved in more than 10 different pathways (Fig. 3b). Other antiviral genes like IFNA1, IFNB1, NFKBIA, and IL6 were found involved in at least 5 different pathways.

Here we propose to use FCM not only to identify viral responsive gene-sets to the influenza infection but also to identify the genes overlapping across different gene-sets. FCM is a soft version of K-means clustering that allows overlap between the gene-sets reproducing the topology of the known pathways. Here we optimized the parameters on DC dataset and validated those on EC dataset (refer to methods). FCM pipeline described in methods led to an average size of gene-sets 167 (standard deviation of 45), with smallest gene-set having 63 and largest gene-set having 230 genes. With this configuration one third of the genes exhibited overlapping behavior where 1943 out of 5789 genes belonged to more than one gene-sets (Fig. 3c and d).

### Validation of FCM Gene-Sets

**Fig. 4:**
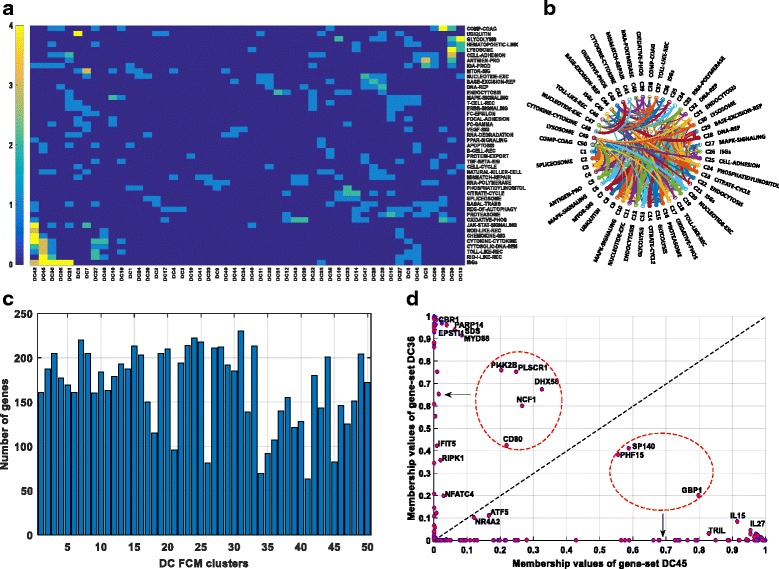
Validation of DC FCM gene-sets. **a** The enrichment of KEGG pathways and ISGs in DC FCM gene-sets, five colors ranging from *blue* to *yellow* represent –log10 (p-value) ≤1.30, >1.30 and ≤3, >3 and ≤4, >4 and ≤5, and >5 calculated by hypergeometric test, **b** Circular graph represents overlap between the DC FCM gene-sets, **c** number of genes in DC FCM gene-sets and **d** membership values of the genes DC36 and DC45, and overlapping genes (*circled in red*) between DC36 and DC45

To assess if gene-sets identified by FCM pipeline indeed grouped the functionally related genes, we compared the FCM-gene-sets with the pathways defined in KEGG and by Schoggins [37, 38]. Schoggins-gene-set defines Interferon Stimulated Genes (ISGs) and has been reported to be significantly enriched upon influenza infections by previous studies [8, 23]. 43 out of 50 FCM-gene-sets were found enriched in at least one of the pathways (*p* value <0.01) (Fig. 4a and b). FCM-gene-sets DC21, DC26, DC36 and DC45 were found significantly enriched with ISGs (*p* values 3.3 *e* ^− 10^, 1.19 *e* ^− 11^, 5.36 *e* ^− 29^ and 1.72 *e* ^− 60^ respectively). Cluster 45 was also found enriched with RIG-I-Like and Toll-Like receptor signaling pathways (*p* values 3.04 *e* ^− 6^ and 1.32 *e* ^− 5^) which are critical pathogen recognition receptor mediated pathways known to be induced upon viral infections [23]. Similarly, gene-set DC42 was enriched with other well-known anti-viral pathways (JAK-STAT, Chemokine and Cytokine-Cytokine signaling pathways (*p* values 4.69 *e* ^− 6^, 1.5 *e* ^− 6^ and 3.22 *e* ^− 16^ respectively)). The enrichment results indeed corroborates with the previously published results validating FCM-gene-sets [20, 23]. Interestingly, there were 7 (gene-sets DC1, DC3, DC4, DC9, DC19, DC34 and DC35) novel sets, which were not significantly enriched in any of the pathways. Most of these gene-sets had genes overlapping with other gene-sets enriched in known pathways, suggesting multi-functionality of the overlapping genes (Additional file 1: Figure S1). Thus, FCM pipeline not only validated previously known functionally related genes but also identified new sets of genes.

### Genes associated with multiple gene-sets are identified by FCM-pipeline

FCM pipeline was developed to find genes that are associated with multiple gene-sets. There were 1943 overlapping genes associated with minimum 2 and maximum 5 gene-sets. Interestingly 113 genes involved in multiple KEGG pathways were also found by our pipeline (Table 1). While involvement of genes across multiple KEGG pathways is not evidence for the multi-functionality of the genes it is the only available data for systematic comparison. Indeed, gene like PIK3R1 involved in 14 pathways (Table 1) could be due to bias in the studies associated with that gene. Genes overlapping between the gene-sets DC45 (82 genes) and DC36 (107 genes) were particularly of interest since both the gene-sets were enriched in anti-viral pathways [23]. 9 genes (GBP1, SP140, PHF15, DHX58, NCF1, PLSCR1, CD80, PI4K2B and NR4A2) were in common between DC45 and DC36 gene-sets, and their membership values ranged from 0.2 to 0.8 (Fig. 4d). Genes closer to gene-set DC45 or gene-set DC36, showed stronger association in the corresponding gene-sets, e.g. DHX58 belonged to gene-set DC36 with membership value of 0.675 and gene-set DC45 with membership value of 0.325 suggesting that DHX58 have a more dominant (67.5%) association with gene-set DC36 and less dominant but considerably significant (32.5%) association with gene-set DC45 (Fig. 4d).

**Table 1:** Comparison of multifunctional genes from FCM gene-sets and KEGG pathways. Multifunctional genes that were involved in at least 3 FCM DC gene-sets were also overlapping between KEGG pathways

Multifunctional genes	No. of pathways	No. of FCM DC clusters	Enriched pathway names	FCM cluster
NFATC4	5	5	MAPK_SIGNALING, VEGF_SIGNALING, NATURAL_KILLER_CELL_MEDIATED_CYTOTOXICITY, T_CELL_RECEPTOR_SIGNALING, B_CELL_RECEPTOR_SIGNALING	34,35,37,43,45
CCL23	2	4	CYTOKINE_CYTOKINE_RECEPTOR_INTERACTION, CHEMOKINE_SIGNALING_PATHWAY	17,18,39,40
GAB2	2	4	FC_EPSILON_RI_SIGNALING, FC_GAMMA_R_MEDIATED_PHAGOCYTOSIS	13,16,19,31
IL21R	2	4	CYTOKINE_CYTOKINE_RECEPTOR_INTERACTION, JAK_STAT_SIGNALING	7,8,20,24
VASP	2	4	FOCAL_ADHESION, FC_GAMMA_R_MEDIATED_PHAGOCYTOSIS	1,4,10,50
ANAPC1	2	3	CELL_CYCLE, UBIQUITIN_MEDIATED_PROTEOLYSIS	7,8,29
ASAP1	2	3	ENDOCYTOSIS, FC_GAMMA_R_MEDIATED_PHAGOCYTOSIS	30,31,39
CCND2	3	3	CELL_CYCLE, FOCAL_ADHESION, JAK_STAT_SIGNALING	7,14,29
CD80	4	3	CELL_ADHESION_MOLECULES_CAMS, TOLL_LIKE_RECEPTOR_SIGNALING_PATHWAY, INTESTINAL_IMMUNE_NETWORK_FOR_IGA_PRODUCTION, ISGs	36,45,46
CDC16	2	3	CELL_CYCLE, UBIQUITIN_MEDIATED_PROTEOLYSIS	8,14,29
CDK4	2	3	CELL_CYCLE, T_CELL_RECEPTOR_SIGNALING	23,33,44
DNM2	2	3	ENDOCYTOSIS, FC_GAMMA_R_MEDIATED_PHAGOCYTOSIS	3,12,31
EP300	3	3	JAK_STAT_SIGNALING, CELL_CYCLE, TGF_BETA_SIGNALING,	3,4,50
HSPB1	2	3	MAPK_SIGNALING, VEGF_SIGNALING	5,25,50
IL1R2	3	3	MAPK_SIGNALING, CYTOKINE_CYTOKINE_RECEPTOR_INTERACTION, HEMATOPOIETIC_CELL_LINEAGE	6,21,22
ITGAV	2	3	FOCAL_ADHESION, CELL_ADHESION_MOLECULES_CAMS	2,25,50
MAP3K1	3	3	MAPK_SIGNALING_PATHWAY, UBIQUITIN_MEDIATED_PROTEOLYSIS, RIG_I_LIKE_RECEPTOR_SIGNALING	1,27,50
POLR1C	2	3	RNA_POLYMERASE, CYTOSOLIC_DNA_SENSING	3,31,33
PPP3CB	6	3	MAPK_SIGNALING, APOPTOSIS, VEGF_SIGNALING, NATURAL_KILLER_CELL_MEDIATED_CYTOTOXICITY, T_CELL_RECEPTOR_SIGNALING, B_CELL_RECEPTOR_SIGNALING	16,28,29
RPS6KB1	4	3	ERBB_SIGNALING, MTOR_SIGNALING, TGF_BETA_SIGNALING, FC_GAMMA_R_MEDIATED_PHAGOCYTOSIS	16,23,28
TNFRSF1A	3	3	MAPK_SIGNALING, CYTOKINE_CYTOKINE_RECEPTOR_INTERACTION, APOPTOSIS	5,25,50
WAS	2	3	CHEMOKINE_SIGNALING, FC_GAMMA_R_MEDIATED_PHAGOCYTOSIS	23,28,44
PIK3R1	14	3	T CELL RECEPTOR SIGNALING, B CELL RECEPTOR SIGNALING, TOLL LIKE RECEPTOR SIGNALING and 11 others	7,8,24

One overlapping gene of a particular interest was CD80, a protein found on monocytes that provides a costimulatory signal necessary for T cell activation and survival. It is a ligand for two different proteins on the T cell surface: CD28 (for auto-regulation and intercellular association) and CTLA-4 [43, 44]. CD80 was associated with gene-sets DC45, DC36 and DC46 suggesting that CD80 has a multifunctional role in induction of several gene-sets. Genes like CD80 are involved in stimulating multiple down-stream events and therefore do not have a strong membership to one particular gene-set. These genes are critical in developing intervention strategies and understanding mechanisms of cross-talk, however, are typically ignored by hard clustering algorithms.

### Gene-sets enriched in ISGs have distinct temporal patterns

**Fig. 5:**
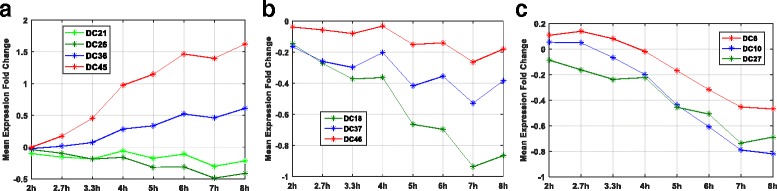
The temporal expression of the gene-sets enriched with KEGG pathways. Mean temporal expression of gene-sets significantly enriched (*p* < 0.01) with **a** ISGs (DC21, DC26, DC36 and DC45), **b** Toll-like receptor signaling pathway (DC18, DC37 and DC46) and **c** MAPK signaling pathway (DC6, DC10 and DC27)

The data-driven clustering in context-specific manner can reveal sets of genes which are functionally diverse even though they are typically grouped together [37, 38]. Specifically, previously known ISGs were grouped into 4 gene-sets (DC21, DC26, DC36 and DC45). Gene-sets DC21 and DC26 were down-regulated with time whereas gene-sets DC36 and DC45 were up-regulated with time (Fig. 5a). The mean temporal expression pattern of gene-set DC26 was different than that of gene-set DC21 (Fig. 5a). Similarly, at any given time, the mean expression of gene-set DC45 was more than twice compared to that of gene-set DC36. Also, gene-sets DC45 and DC26 were more steeply up and down regulated as compared to the gene-set DC36 and DC21 respectively. Previously, time delays have been used to infer regulatory relationships [45] suggesting that gene-set DC45 might regulate gene-set DC36 and gene-set DC26 might regulate gene-set DC21. Similarly, other clusters (Fig. 5b and c) that were enriched with same pathway showed differences in the magnitude of gene expression, rate of activation and sign of mean expression.

### FCM clustering is flexible and comparable to other widely used clustering methods

**Fig. 6:**
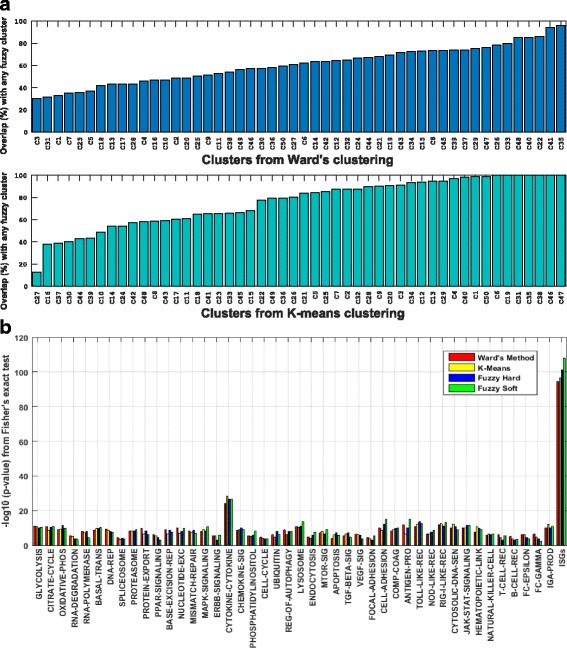
Comparison of FCM with hard clustering methods. **a** Number of genes overlapping between FCM gene-sets and k-means with Ward’s initialization (*bottom*), and Ward’s hierarchical clustering (*top*) and **b** the enrichment of ISGs and KEGG pathways﻿ by Fisher's exact tes﻿t in clusters identified by K-mean, ﻿hierarchichal and FCM methods

The comparison of FCM with commonly used algorithms such as k-means and hierarchical clustering using Ward’s method yielded comparable results. Both FCM and K-means clustering were performed by optimizing initial cluster centers by Ward’s method. Genes from FCM solution were associated with a unique cluster (one with which a gene has a maximum membership value) thus producing hard clusters that can be compared to the solution of k-means and hierarchical clustering algorithms. Cluster sizes, mean node degrees, mean local CCs and mean global CCs were compared for the assessment of cluster quality. K-means, hierarchical clustering and FCM produced 45, 44 and 44 clusters respectively that had higher local CC than the global CC indicating the identification of a comparable number of cohesive clusters. K-means and hierarchical clusters had a minimum of 13% and 30%, and a maximum of 100 and 96% respective overlap with FCM clusters (Fig. 6a). This suggests that K-means, Ward’s hierarchical method and FCM were able to pick fundamental characteristics of gene expression data. Additionally, enrichment of KEGG pathways and ISGs in the clusters from all three methods suggested that ISGs and genes involved in Cytokine-Cytokine receptor signaling pathways robustly cluster together (Fig. 6b). In conclusion, FCM is not only comparable with other clustering methods but also facilitate identification of genes with the possible multi-functional role.

### Application of FCM to other cell-types

**Fig. 7:**
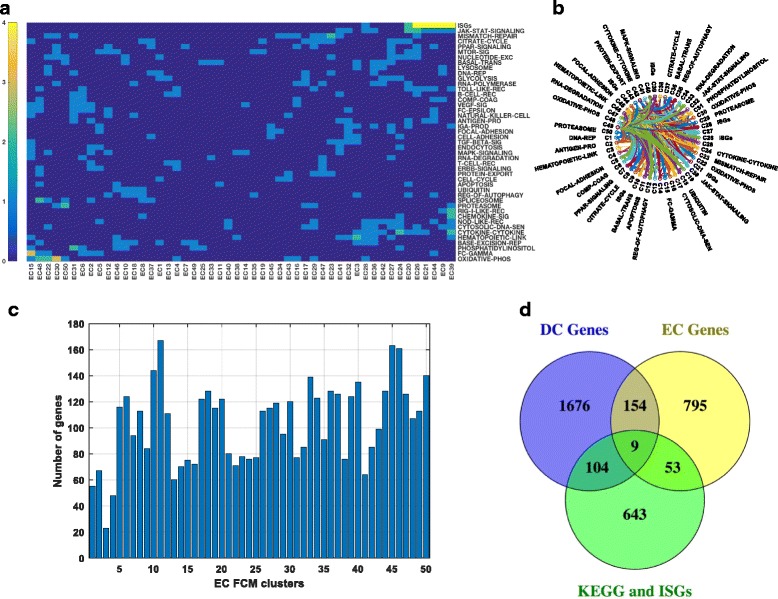
Application of FCM pipeline on EC dataset. **a** The enrichment of KEGG pathways and ISGs in EC FCM gene-sets, five colors ranging from *blue* to *yellow* represent –log10 (p-value) ≤1.30, >1.30 and ≤3, >3 and ≤4, >4 and ≤5, and >5 calculated by hypergeometric test, **b** Circular graph represents overlap between the EC FCM gene-sets, **c** number of genes in 50 EC FCM gene-sets, and **d** Venn diagram representing number of genes overlapping between at least two FCM gene-sets in DC, EC, and KEGG/ISGs pathways

ECs and DCs are early responders to the viral infections, which signal through pathogen recognition receptor induced pathways. Comparison of genome-wide gene-expression profiles across two cell-types reveals a small overlapping sub-network and a large cell-specific response to influenza infections [20]. Application of FCM pipeline to EC dataset revealed 34% (1298) of overlapping genes and significant enrichment of several KEGG pathways and ISGs in 39 out of 50 EC gene-sets (Fig. 7a and b). 167 overlapping genes were common in EC and DC (Fig. 7d), and 9 overlapping genes (PYCARD, ATP6V1H, ENO1, HSPA1A, PTPN11, CCNH, CSF1, CXCL2 and HK2) were common in DCs, ECs and also in KEGG pathways (Fig. 7d). In conclusion, FCM can be robustly applied to different cell-specific transcriptomic data to identify overlapping genes.

### Development of FIGS: a Fuzzy Inference of Gene-sets package

The power of GSEA-like test will be improved by using robust context-specific gene-sets. To facilitate the use of computational model presented in this study we developed a Matlab-based installable package called ‘Fuzzy Inference of the Gene-sets (FIGS)’ (available at https://github.com/Thakar-Lab/FIGS). This package can be used to obtain gene-sets from matrix defining the pair-wise distance between the genes. FIGS also provide an option to upload pathways for enrichment analysis of gene-sets. FIGS package requires three parameters: number of clusters, fuzziness allowed between the clusters, and cluster association criteria to produce fuzzy gene-sets. Once the number of clusters and the amount of overlap between the clusters (fuzziness) is defined, the user has four different choices for associating genes to the clusters: 1) genes are assigned to a unique cluster based on their highest degree of membership, 2) distribution based association method described and used in this manuscript, 3) cluster with membership value higher than mean of the maximum membership values, and 4) user defined threshold (between 0-1). The results are stored in tabular form and are also displayed as interactive circular graphs. Other functionalities are described in the user’s manual. For those interested in exploring or using the gene-sets produced from the meta-analysis of transcriptomics response of dendritic cells and epithelial cells to influenza infection can access FIGS-Influenza package at https://github.com/Thakar-Lab/FIGS-Influenza. In FIGS-Influenza users can upload their differentially expressed genes or genes of interest for enrichment across fuzzy clusters.

## Discussion

Unsupervised clustering of genome-wide gene expression data is a frequently used tool to identify genes with similar patterns across treatments and/or time-points. We and others have frequently used hierarchical clustering algorithm to identify such groups of genes [20, 41]. Chaussabel et. al. introduced a concept of modules which are derived using K-means clustering and can be used as a set of a priori defined genes in pathway analysis [9, 10]. However, these hard clustering algorithms do not fully reproduce the observed topology of the biological pathways. Specifically, all public repositories of the biological pathways share genes across multiple pathways indicating diversity in the functional roles of these genes. Here we present a soft clustering technique to identify gene-sets with overlapping genes that reproduce the characteristics of the pathways in the public repositories and define robust gene-sets by meta-analysis.

We present a pipeline using FCM which has been optimized for cell-specific transcriptomic studies. The integration of multiple context-specific datasets provides more robust and universal gene-sets as compared to the FCM performed on individual data set. FCM parameters optimized in this study are based on the distribution of cluster association values. The upper bound of fuzziness values (m) and the distribution based cluster association have been suggested previously but never used for gene-gene association networks [28]. Additionally, our fuzziness selection criteria, selection of robust initial centroids by Ward’s method and validation of the clustered gene-sets is extremely relevant to human immunology studies. Interestingly, FCM pipeline developed here produced gene-sets that were concise and robust compared to previously defined criteria for inference of gene-sets for pathway analysis [46].

FCM pipeline proposed here will improve the data-driven inference of gene-sets by two ways. First, by identifying overlapping genes that span across multiple gene-sets. These multi-functional genes have diverse roles in signal transduction (e.g. CCL23) and cross-talk between different pathways (e.g. MAP3K1 and GAB2) (Table 1). Thus, in addition to assessing activities of gene-sets by gene-set enrichment method, separate evaluation of multi-functional genes connected to the enriched gene-sets will improve follow-up experiments required to provide mechanistic insights. Second, connecting different gene-sets through the multi-functional genes will improve interpretation of gene-sets that are not enriched in known biological processes. Thus, FCM pipeline will significantly increase the number of novel pathways studied followed by high-throughput omics experiments. In conclusion, the results show that the FCM pipeline recapitulates topological characteristics of the biological pathways and will improve data-interpretation required for follow-up experiments.

We adapted Fuzzy-C-Means clustering algorithm, which is similar to previously used K-means clustering algorithm [9, 10], but in addition allows identification of the genes with functional roles across more than one cluster. One reason for the limited use of FCM in transcriptomic studies is the difficulty in optimizing the FCM parameters and initial centroids. Unlike previously suggested method of assigning centroids using prior biological knowledge [47] we use Ward’s method. The Ward’s method used in our study infers robust clusters. Moreover, our previous analysis shows that genes defined by the prior biological knowledge do not always form cohesive clusters leading to erroneous clustering solutions. Additionally, parameters optimized by the previous applications of FCM for yeast transcriptomic data cannot be applied to the transcriptomic data generated from humans [28, 48–51].

Use of gene-sets derived from context-specific transcriptomic data in the public domain will enhance the ability to develop hypotheses from high-throughput experiments. Cell-type is one of the critical contexts for all immunological studies and here we propose the FCM pipeline that can be applied to different cell-types. However, our previous study reveals that gene-gene associations inferred from cell-specific independent experiments are more robust than a mixture such as peripheral blood monocytes (PBMCs) [20]. Thus, even though FCM parameters optimized here could be applied to two different cell-types; it is likely that the parameters of FCM will need to be characterized separately for PBMC datasets.

In future the proposed pipeline will be applied to transcriptomic data measuring cell-type specific responses to the stimuli, purified proteins or viruses, and FIGS package will be expanded to include these results.

## Conclusions

In this study we develop a pipeline using Fuzzy-C-Means clustering algorithm to identify multi-functional genes from meta-analysis of context-specific transcriptomic datasets. Additionally, the approach proposed here reveals novel gene-sets and facilitates their interpretation. Moreover, delivery of our pipeline by interactive FIGS package (https://github.com/Thakar-Lab/FIGS) will increase the accessibility and usability of the data-driven context-specific gene-sets in future studies.

## Additional file

Additional file 1: Figure S1. FCM pipeline facilitates functional interpretation of novel DC gene-sets. FCM DC gene-sets without enrichment of the immunological pathways (DC1, DC3, DC4, DC9, DC19, DC34 and DC35) were associated with gene-sets enriched in known-pathways facilitating interpretation of novel gene-sets. (PPTX 184 kb)

## Abbreviations

CC: Clustering coefficient
DC: Dendritic cell
EC: Epithelial cell
FCM: Fuzzy c-means clustering
FIGS: Fuzzy inference of gene-sets
ISG: Interferon stimulated genes
KEGG: Kyoto encyclopedia of genes and genomes
MI: Mutual information
PBMC: peripheral blood monocytes
PCA: Principal component analysis
