# Supplementary figures and images for: Meta-analysis of cell- specific transcriptomic data using fuzzy c-means clustering discovers versatile viral responsive genes
Source: BMC Bioinformatics. 2017 Jun 6;18:295. doi: 10.1186/s12859-017-1669-x (PMC5461682; doi:10.1186/s12859-017-1669-x)

## Slide 1
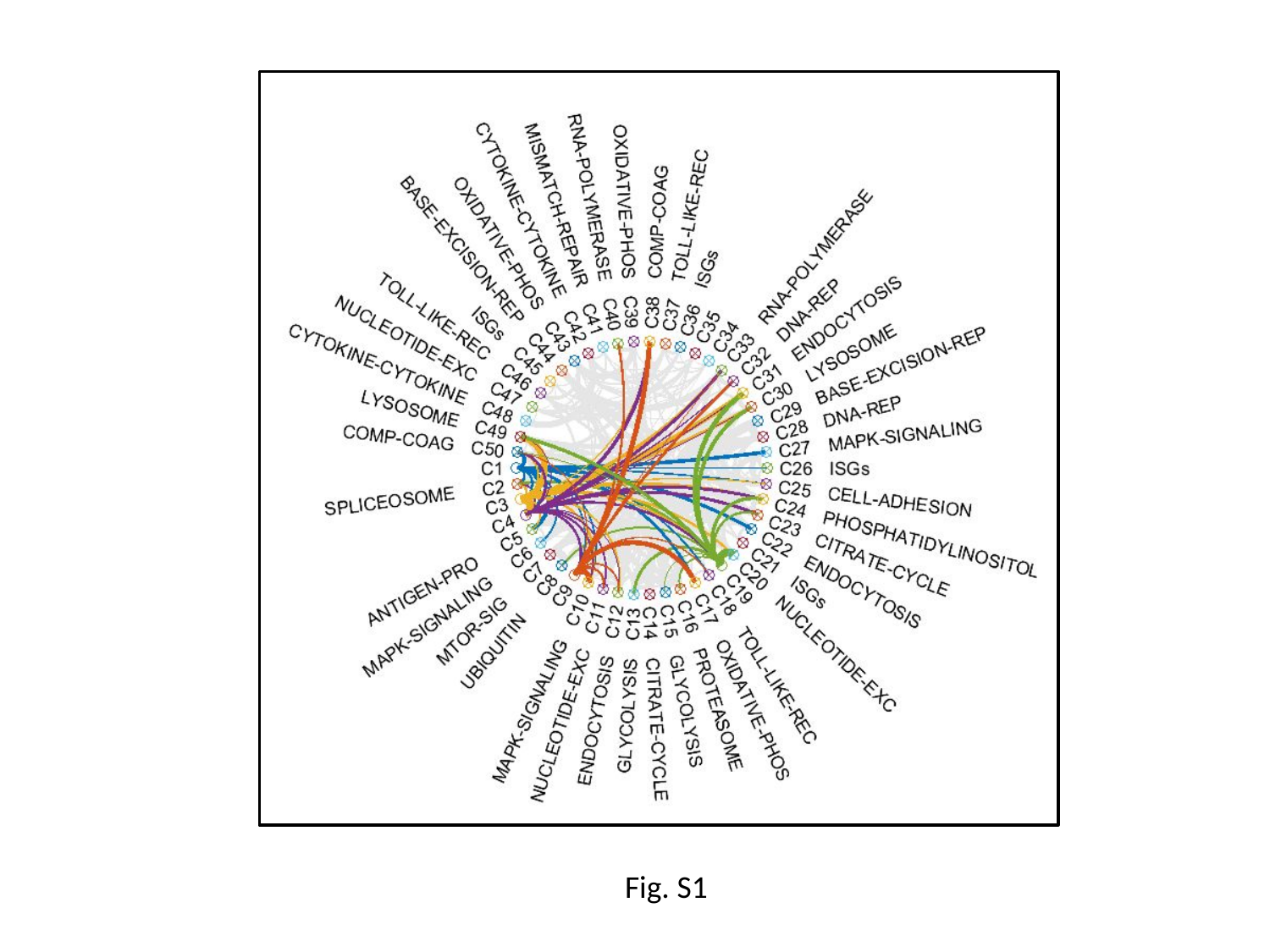

Fig. S1

Supplement: Additional file 1: Figure S1. — FCM pipeline facilitates functional interpretation of novel DC gene-sets. FCM DC gene-sets without enrichment of the immunological pathways (DC1, DC3, DC4, DC9, DC19, DC34 and DC35) were associated with gene-sets enriched in known-pathways facilitating interpretation of novel gene-sets. (PPTX 184 kb) [file 12859_2017_1669_MOESM1_ESM.pptx]
